# Prophylactic central neck dissection and local recurrence in papillary thyroid microcarcinoma: a meta-analysis^☆^^☆☆^

**DOI:** 10.1016/j.bjorl.2018.05.004

**Published:** 2018-06-20

**Authors:** Hui Su, Yujie Li

**Affiliations:** Ningbo No. 2 Hospital, Department of Surgical Oncology, Ningbo, China

**Keywords:** Central neck dissection, Local recurrence, Papillary thyroid microcarcinoma, Meta-analysis, Esvaziamento cervical central, Recidiva local, Microcarcinoma papilífero de tireoide, Metanálise

## Abstract

**Introduction:**

For papillary thyroid microcarcinoma patients, the reported incidence of lymph node metastasis is as high as 40%, and these occur mainly in the central compartment of the neck. Because these metastases are difficult to detect using ultrasonography preoperatively, some authors advocate routine central neck dissection in papillary thyroid microcarcinoma patients at the time of initial thyroidectomy.

**Objective:**

To evaluate whether prophylactic central neck dissection can decrease the local recurrence rate of papillary thyroid microcarcinoma after thyroidectomy.

**Methods:**

The publicly available literature published from January 1990 to December 2017 concerning thyroidectomy plus prophylactic central neck dissection versus thyroidectomy for papillary thyroid microcarcinoma was retrieved by searching the national and international online databases. A meta-analysis was performed after the data extraction process.

**Results:**

Four studies were finally included with a total of 727 patients, of whom, 366 cases underwent thyroidectomy plus prophylactic central neck dissection and 361 cases received thyroidectomy only. As shown by the meta-analysis results, the recurrence rates in cases of thyroidectomy plus prophylactic central neck dissection were approximately 1.91% and were significantly lower than those with thyroidectomy only (OR = 0.24, 95% CI [0.10, 0.56], *p* = 0.0009).

**Conclusion:**

For patients with papillary thyroid microcarcinoma, thyroidectomy plus prophylactic central neck dissection is a safe and efficient procedure and it results in lower recurrence rate. Since the evidences are of low quality (non-randomized studies), further randomized trials are needed.

## Resumo

**Introdução:**

A incidência relatada de metástases linfonodais chega a 40% em pacientes com microcarcinoma papilífero de tireoide e essas ocorrem principalmente no compartimento cervical central. Como essas metástases são difíceis de ser detectadas com o uso de ultrassonografia no pré-operatório, alguns autores defendem o esvaziamento cervical central de rotina em pacientes portadores de microcarcinoma papilífero de tireoide no momento da tireoidectomia inicial.

**Objetivo:**

Avaliar se o esvaziamento cervical central profilático pode diminuir a taxa de recorrência local de microcarcinoma papilífero de tireoide após a tireoidectomia.

**Método:**

A literatura disponível, publicada de janeiro de 1990 a dezembro de 2017, sobre tireoidectomia com esvaziamento cervical central profilático versus tireoidectomia somente para microcarcinoma papilífero de tireoide foi obtida através de busca nas bases de dados online nacionais e internacionais. A metanálise foi feita após o processo de extração de dados.

**Resultados:**

Quatro estudos foram finalmente incluídos na metanálise, com 727 pacientes, dos quais 366 foram submetidos à tireoidectomia com esvaziamento cervical central profilático e 361 só receberam tireoidectomia. Como mostrado pelos resultados da metanálise, as taxas de recorrência com tireoidectomia com esvaziamento cervical central profilático foram de 1,91% e foram significantemente menores do que aquelas em pacientes submetidos somente à tiroidectomia (OR = 0,24, IC95% [0,10-0,56], *p* = 0,0009).

**Conclusão:**

Para pacientes com microcarcinoma papilífero de tireoide, o esvaziamento cervical central profilático é um procedimento seguro e eficiente e resulta em menor taxa de recorrência. Como as evidências são de baixa qualidade (estudos não randomizados), mais estudos randomizados são necessários.

## Introduction

Papillary thyroid microcarcinoma (PTMC) is defined as a papillary thyroid carcinoma that is equal to or less than 1.0 cm at the greatest dimension according to the World Health Organization classification system for thyroid tumors.^1^ The majority of PTMCs are not palpable and clinically inapparent.^2^ For PTMC patients, the reported incidence of lymph node metastasis is as high as 40%, and these occur mainly in the central compartment of the neck.^3^, ^4^, ^5^ Because these metastases are difficult to detect using ultrasonography preoperatively, some authors advocate routine Central Neck Dissection (CND) in PTMC patients at the time of initial thyroidectomy.^6^, ^7^, ^8^, ^9^ The purpose of this study is to evaluate the influence of CND on the postoperative complications and recurrence of patients with PTMC.

## Materials and methods

### Search strategy

PubMed, Web of Knowledge, Ovid's database were searched from January 1990 to December 2017 with English language. The search terms used were “thyroidectomy”, “central neck dissection”, “local recurrence” and “papillary thyroid microcarcinoma”. The reference lists of relevant studies were checked manually to locate any missing studies.

### Study selection

Identified studies were assessed for eligibility for inclusion in the review by scrutinizing the titles, abstracts and keywords of every record retrieved. Studies were restricted to those published in English. Clinical studies concerning comparisons of any aspects between the CND+ group and CND− group for PTMC were also included.

### Data extraction

Two coauthors (LY and SH) independently assessed the methodological quality of each study using the Methodological Index for Non-Randomized Studies criteria (MINORS).^10^ The following variables were recorded: authors, journal and year of publication, number of patients, age, transient RLN palsy, permanent RLN palsy, transient hypoparathyroidism, permanent hypoparathyroidism and recurrence. If necessary, the corresponding authors of studies were contacted to obtain supplementary information.

### Statistical analysis

A formal meta-analysis was carried out for all included studies comparing the results of CND+ and CND− for PTMC. The outcomes in our study were transient RLN palsy, permanent RLN palsy, transient hypoparathyroidism, permanent hypoparathyroidism and recurrence. A fixed effects model was used to calculate a pooled Odds Ratio (OR) with its 95% confidence interval (CI). Heterogeneity was explored using *I*^2^ statistics, a measure of how much the variance between studies, rather than chance, can be attributed to inter-study differences. *I*^2^ > 50% was regarded to indicate strong heterogeneity. The Cochrane Collaboration's Review Manager Software (RevMan version 5.0) was utilized for the data analysis.

## Results

### Study selection

**Figure 1:**
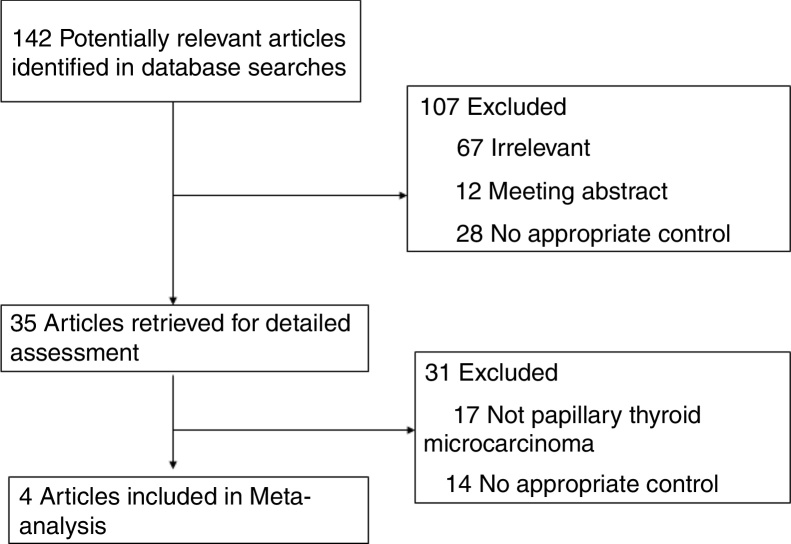
Flowchart of the results of the literature search.

We identified 142 potentially relevant articles (Fig. 1). After exclusion of duplicate references, non-relevant literature, and those that did not satisfy inclusion criteria, 35 candidate articles were considered for the meta-analysis. After careful review of the full text of these articles, 4 studies were included. The study characteristics were summarized in Table 1.

**Table 1:** Overview of the reviewed studies.

Author, year	Country	No. of patients	Sex (male/female)	Patient source	Mean age	Study design
Hyun et al., 2012^11^	Korea	152	CND+: 9/56 CND−: 20/67	University of Ulsan	CND+: 46 CND−: 48	Retrospective
Choi et al., 2008^12^	Korea	101	CND+: 6/42 CND−: 11/42	University of Ulsan	CND+: 52 CND−: 48	Retrospective
Zhang et al., 2015^13^	China	242	CND+: 26/108 CND−: 27/81	Peking Union Medical College Hospital,	CND+: 48 CND−: 45	Retrospective
So et al., 2012^14^	Korea	232	CND+: 98/21 CND−: 97/16	Sungkyunkwan University School of Medicine,	CND+: 49.18 CND−: 49.75	Retrospective

CND−, total thyroidectomy (TT) alone/hemithyroidectomy alone; CND+, TT/hemithyroidectomy plus central lymph node dissection.

Patient demographics for the 4 studies are presented in Table 1. All papers were retrospective clinical trials. The publication dates ranged from January 1990 to December 2017. Study sizes ranged from 101 to 242 patients. The assessments of the non-randomized studies are illustrated in Table 2. The median quality score was 12.5.

**Table 2:** Assessment of the quality of the studies using the methodological index for non-randomized studies (MINORS).

Author, year	A clearly stated aim	Inclusion of consecutive patients	Prospective collection of data	Endpoints appropriate to the aim of the study	Unbiased assessment of the study endpoint	Follow-up period appropriate to the aim of the study	Loss to follow up less than 5%	Prospective calculation of the study size	Score
Hyun et al., 2012	2	2	2	2	2	1	2	0	13
Choi et al., 2008	2	2	2	1	1	1	2	0	11
Zhang et al., 2015	2	2	2	2	1	2	2	0	13
So et al., 2012	2	2	2	2	2	1	2	0	13

0, represented that the item was not reported in the article; 1, represented that the item was reported but deficiently; 2, represented that the item was reported completely and appropriately.

### Outcome measures

A total of 366 patients who underwent CND+ and 361 patients who underwent CND− were analyzed. The criteria for the temporary/permanent hypocalcemia, transient Recurrent Laryngeal Nerve (RLN) palsy and recurrences were summarized in Table 3.

**Table 3:** The criteria for the complications and recurrences.

Author, year	The criteria used for temporary hypocalcemia	The criteria used for permanent hypocalcemia	The criteria used for temporary RLN palsy	The criteria used for permanent RLN palsy	The criteria used for the recurrences
Hyun et al., 2012	–	–	–	–	–
Choi et al., 2008	The need for exogenous calcium replacement in order to maintain a normal range of serum total calcium (8–10.4 mg/dL) or to eliminate the clinical signs and symptoms of hypocalcemia	Calcium replacement was necessary for longer than 12 months	–	–	Confirmed by ultrasonography-guided fine needle aspiration cytology
Zhang et al., 2015	Serum calcium <8 mg/dL anytime during the initial 6-month follow-up	A need for continued calcium beyond 6 months after surgery with persistent serum calcium <8 mg/dL	By fiber optic laryngoscopy between 0 and 6 months after operation	Confirmed by fiber optic laryngoscopy beyond 6 months after operation	Detected by serial cervical ultrasonographies or radioactive thyroid scan
So et al., 2012	At least 1 event of hypocalcemic symptoms (perioral numbness, paresthesias of the hands and feet, Chvostek sign, and Trousseau sign) or at least 1 event of biochemical hypocalcemia (ionized Ca level <1.0 mmoL/L)	Persistent symptoms or persistent biochemical hypocalcemia greater than duration of 6 months.	Checked with a ﬁberoptic ﬂexible laryngoscope or a rigid telescopic laryngoscope.	–	–

**Figure 2:**
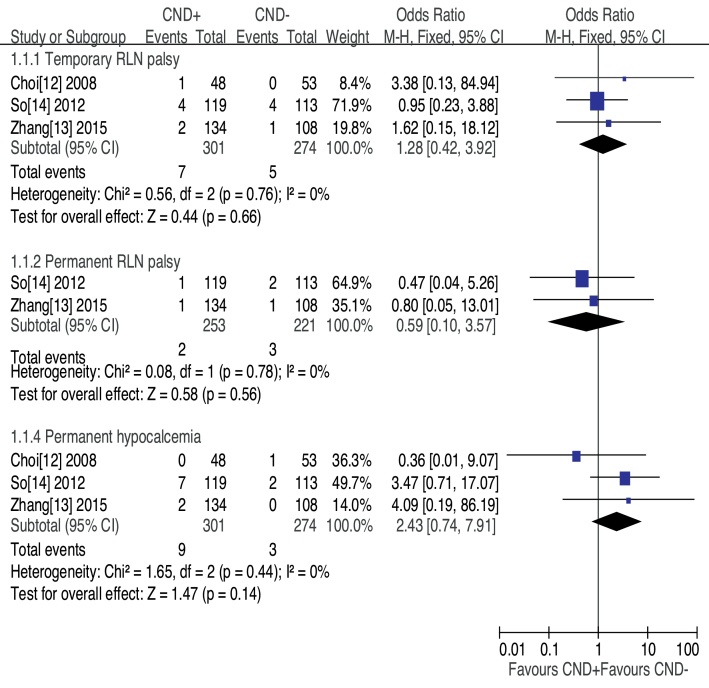
Forest plot of the comparison of temporary RLN palsy and permanent hypocalcemia for CND+ vs. CND−.

**Figure 3:**
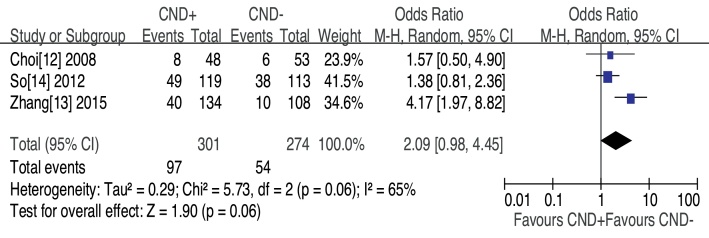
Forest plot of the comparison of temporary hypocalcemia for CND+ vs. CND−.

Transient recurrent laryngeal nerve palsy was observed in three studies, CND− group had less transient RLN palsy, but no significant difference was found (OR = 1.28, 95% CI [0.42–3.92], *p* = 0.66) (Fig. 2). The prevalence of permanent RLN palsy was 0.79% in the CND+ group vs. 1.36% in the CND− group without significant difference (OR = 0.59, 95% CI [0.10–3.57], *p* = 0.56) (Fig. 2). Three studies assessed patients for transient hypocalcemia. The prevalence of transient hypocalcemia was 32.23% in the CND+ group vs. 19.71% in the CND− group, and this difference was not significant (OR = 2.09, 95% CI [0.98–4.45], *p* = 0.06) (Fig. 3). The prevalence of permanent hypocalcemia was 2.99% in the CND+ group vs. 1.09% in the CND− group, and no significant difference was observed (OR = 2.43, 95% CI [0.74–7.91], *p* = 0.14) (Fig. 2).

**Figure 4:**
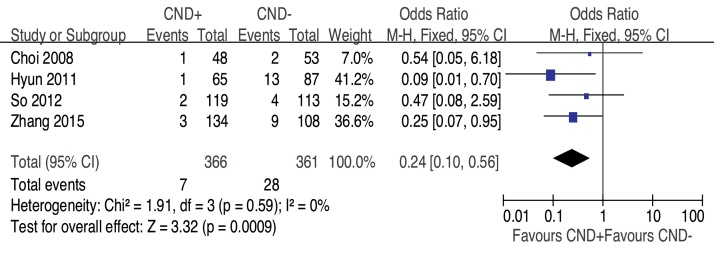
Forest plot of the comparison of recurrence for CND+ vs. CND−.

Recurrence was assessed in all four studies. The recurrence rates in CND+ were approximately 1.91% and were significantly lower than those in CND− (OR = 0.24, 95% CI [0.10, 0.56], *p* = 0.0009) (Fig. 4).

## Discussion

Higher rates of complications such as temporary hypocalcemia, permanent hypocalcemia, and RLN palsy are often cited in arguments against prophylactic CND in the treatment of PTMC.^15^, ^16^ Temporary hypocalcemia has been reported to be between 20% and 50%.^17^, ^18^, ^19^ In our meta-analysis, the incidence of temporary and permanent hypocalcemia had no difference between the two groups, suggesting that dissection of the central neck compartment did not enhance damage to the parathyroid glands. Similarly, the rates of temporary and permanent RLN injury did not increase with prophylactic CND.

Some studies reported that the role of CND in PTMC remains uncertain because no evidence has demonstrated that CND improves locoregional control or survival in PTMC.^20^, ^21^ Wada et al.^2^ compared the recurrence rate of 235 patients with PTMC who underwent prophylactic neck dissection with that of 155 patients with incidental PTMC who did not undergo neck dissection. After a 60 month follow-up, the recurrence rate was 0.43% for the dissection group and 0.65% for the non-dissection group. No statistical significance was observed. In addition, Appetecchia et al.^22^ do not believe that CND is necessary, because the reported mortality rates of PTMC range from 0% to 1%, and CND provides no survival benefit. However, the recurrence rates in CND+ were significantly lower than those in CND− in our meta-analysis. Shen et al. have shown a similar trend toward decreased recurrence in patients undergoing prophylactic CND.^23^

On the other hand, the incidence of central lymph node metastases (CLNMs) are relatively common in PTMC patients. Lymph node dissection is generally indicated when there is cervical lymphadenopathy detected either preoperatively or intraoperatively. In this case, central lymph node dissection should be performed at the time of thyroid surgery since subsequent surgery for node metastases in the neck may be technically difficult. However, the effect of prophylactic lymph node dissection on patients without preoperative or intraoperative lymphadenopathy has been disputed.^24^ Currently, the diagnostic performance of Ultrasonography (US) for determining the presence of CLNM in PTMC patients is not completely reliable. The sensitivity of US in predicting CLNM for PTMC patients has been reported to range from 21.6% to 38.0%.^6^, ^25^, ^26^ Several studies have demonstrated that CLNMs are observed in about 31%–64.1% of patients with PTMC.^22^, ^27^, ^28^ Simpson et al.^29^ reported two cases of PTMC that both measured less than 1.5 mm with regional lymph node metastasis and with histological features of regression. In our included studies, the incidence of CLNMs in patients with PTMC was 29.2%–40%.^11^, ^12^, ^13^ We recommend prophylactic central compartment dissection at the time of thyroidectomy. This recommendation is in line with a previous report.^30^

In summary, our meta-analysis demonstrated that there was no increased morbidity in CND+ group. Compared with thyroidectomy alone, combined prophylactic CND may decrease the local recurrence rate. However, the present study has some limitations. First, selection bias is the domain that could lead to a biased estimate of the procedural effects in this analysis. Second, the present study may have been limited by its retrospective non-randomized design. Third, the decision to perform a CND may have been skewed by the surgeon's preference.

## Conclusions

Compared with CND− group, combined prophylactic CND and thyroidectomy is a safe and efficient procedure. It not only excises the occult central lymph node metastases, but also results in lower local recurrence rate of papillary thyroid microcarcinoma. Since the evidences are of low quality (non-randomized studies), further randomized trials are needed.

## Conflicts of interest

The authors declare no conflicts of interest.
